# An isolated population reveals greater genetic structuring of the Australian dingo

**DOI:** 10.1038/s41598-022-23648-1

**Published:** 2022-11-09

**Authors:** Danielle Stephens, Peter J. S. Fleming, Emma Sawyers, Tim P. Mayr

**Affiliations:** 1 Zoological Genetics, Inglewood, SA 5133 Australia; 2 Vertebrate Pest Research Unit, NSW Department of Primary Industries, 1447 Forest Road, Orange, NSW 2800 Australia; 3 Ecosystem Management, School of Environmental and Rural Science, University of New England, Armidale, NSW 2351 Australia; 4 Institute for Agriculture and the Environment, Centre for Sustainable Agricultural Systems, University of Southern Queensland, Toowoomba, QLD 4350 Australia; 5 Vertebrate Pest Research Unit, NSW Department of Primary Industries, 10 Valentine Ave, Parramatta, NSW 2150 Australia; 6 Department of Environment, Land, Water and Planning, 308-390 Koorlong Ave, Irymple, VIC 3498 Australia

**Keywords:** Conservation biology, Invasive species, Molecular ecology

## Abstract

The Australian dingo is a recent anthropogenic addition to the Australian fauna, which spread rapidly across the continent and has since widely interbred with modern dogs. Genetic studies of dingoes have given rise to speculation about their entry to the continent and subsequent biogeographic effects, but few studies of their contemporary population structure have been conducted. Here we investigated the dingo ancestry and population structure of free-living dogs in western Victoria and contrasted it with a wider southern Australian sample. We wished to determine whether their geographic isolation was mirrored in genetic isolation. To address this question, we analysed 34 microsatellite markers using Bayesian clustering and discriminant analysis of principal components, and summarised genetic diversity at the population and individual level. The broader southern Australia sample (n = 1138) comprised mostly hybrid animals, with 30% considered pure dingoes. All western Victorian individuals (n = 59) appeared to be hybrids with high dingo ancestry. The population showed no evidence of admixture with other populations and low genetic diversity on all measures tested. Based upon our characterisation of this unusual mainland population, we advise against assuming homogeneity of dingoes across the continent.

## Introduction

Australia has a large and widely distributed population of free-ranging, wild-living dogs. These animals are all members of the species *Canis familiaris*^1,2^ and include the ancient pure-bred Australian dingoes, (very rarely) stray or feral modern domestic dog breeds, and a swarm of hybrids (≡ cross-breeds) with varying dingo and modern domestic dog lineage^3,4^. These are collectively called “wild dogs” in much of Australian legislation, policy and management strategies^5^.

Australian dingoes are medium sized (~ 15.5 kg^6^), ancient dogs that were most likely derived initially through passive or unconscious domestication from commensal wolves^7^ and later by active domestication of resultant tamer proto-dogs somewhere in southern east Asia about 30,000 years before present^8^. The dingo was introduced to Australia by humans via South East Asia at least 3500–8000 years before present, according to archaeological and molecular evidence respectively^9,10^, and subsequently feralised^6,11^. Corbett^6^ reported trade between north Australian First Peoples and Malaysian seafarers bearing on-board dogs continuing until the 1920s. Although cultural and trade interactions with New Guinea across the Torres Strait persisted, dingoes remained relatively isolated from other dog populations until British colonisation in 1788 (The possible sequence of dingo evolution, introduction in Australia and adaptation can be found in Balme and O’Connor^12^ and Cairns^13^). Being a highly mobile and tameable generalist carnivore, and useful to First Nations peoples, the dingo likely spread from northern Australian introduction points and became established in most Australian environments^6^.

Dingoes and dingo-modern domestic dog hybrids are now widely distributed across most of Australia but their abundance and distribution has been reduced in the south east of the continent^14,15^. Despite advances in the understanding of broad genetic patterns in Australian free-ranging dogs^3,16,17^ since dingoes were first genetically distinguished from hybrids and other dogs^18^, there is still much work to be done disentangling the finer scale patterns that shape local dog populations within the diversity of Australian habitats.

Due to hybridisation between dingoes and modern domestic dogs since 1788 many populations are now hybrid swarms (sensu Allendorf et al*.*^19^) with pure dingoes being more common in central and western Australia, away from dense human settlement^3^. As early agriculturalists took their livestock away from the first British settlements on the coast, lethal control of dingoes began through much of the south east to protect the growing sheep and longhorn cattle industry^20^. The south-eastern mainland state of Victoria was settled early for agricultural pursuits including sheep production, along with New South Wales and south east South Australia^20,21^. Subsequently, dingoes were largely extirpated from Victoria except for the rugged north east, Gippsland in the south east, and the semi-arid far western regions (i.e. the Mallee and Wimmera). There are few pure or possibly pure dingoes in the north east and eastern populations^3^ but the status of the extant western Victorian and its contiguous South Australian population extending to the west (hereafter WV) has not been investigated.

Recent studies^4,11,22^ have examined the genetics of dingo populations with the objectives of informing high level differentiation and identifying possible routes of dingo entry into Australia. Haplotype analysis of dingoes from across Australia showed distinct mitochondrial lineages in south-eastern Australia and K’gari (Fraser Island) and another in north-western and central Australia^22^. Using single nucleotide polymorphisms (SNPs), analysis of 23 samples indicated that the K’gari population was distinct from the south-eastern Australian sample (i.e. “Australian Alpine” dingoes)^4^. The region of the proposed distinction between the south-eastern and north-western lineages has not been well sampled in any previous study. Continued sampling of the intermediate zone between the two lineages is required to determine the point of division; if the lineages are to be treated as separate management units, any relationship of the WV population to the Alpine or other divergences requires assessment.

Although the free-ranging populations in eastern Victoria exhibit local clusters of high dingo ancestry, the majority have evidence of modern dog introgression, which is also evident in contiguous eastern New South Wales populations^3,17^. As in other parts of Australia the prevalence of feral domestic dogs appears to be inconsequential^3^. In this paper we investigated the level of dingo ancestry and population structure of a geographically isolated population of free-living dogs in western Victoria and contiguous South Australia. Our aims were to test for genetic isolation from or continuity with populations identified in previous studies^4,11^.

## Results

### Dingo ancestry

**Figure 1:**
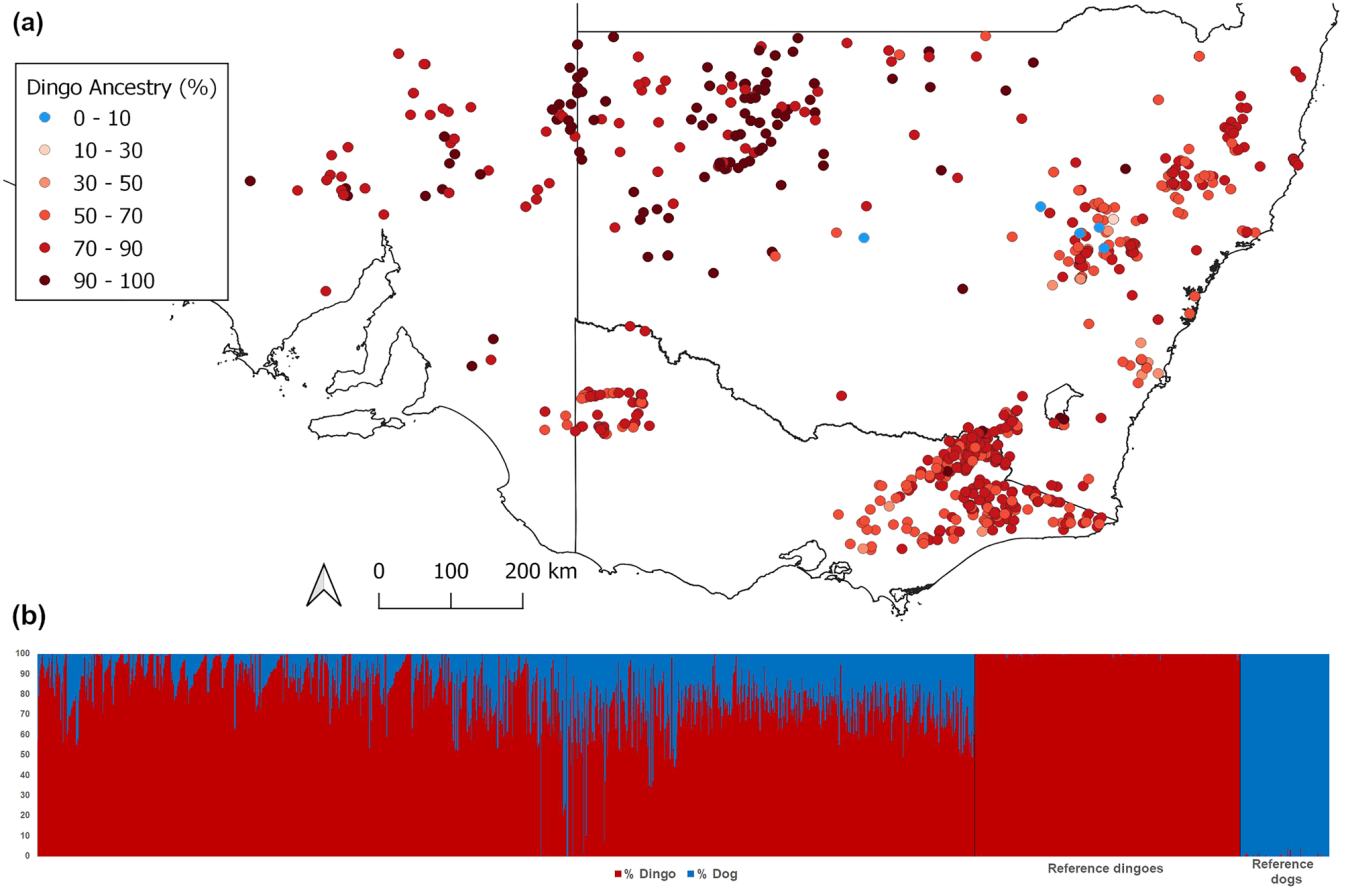
Spatial distribution of free-ranging dogs in south eastern Australia showing higher dingo ancestry in the north west and more hybrids in the south and east (**a**) map of individuals’ dingo ancestry percentage. (**b**) Bar plot of Structure results, sorted by latitude (west to east) then by longitude (north to south) for test individuals. Reference individuals are unsorted. Each line represents the percentage of one individual’s ancestry assigned to either the dingo or domestic dog population.

Dingo ancestry testing was performed using the learning samples approach in Structure (detailed in the Structure v2 documentation), which assigns test individuals to one of two populations (dingoes or domestic dogs) by updating the allele frequencies from assigned reference individuals from each group. Twenty-three microsatellite loci established for allele frequency differences between dingoes and domestic dogs^18^ were used for testing. Details of the reference populations are provided in Stephens et al.^3^. Dingo ancestry testing identified seven individuals (0.6%) with ≤ 10% dingo DNA present (considered modern domestic dogs), 340 (29.9%) individuals with ≥ 90% dingo DNA (considered likely to be pure dingoes) and the remaining 791 (69.5%) individuals were likely hybrids with 11–89% dingo DNA (Table 1). In general, there were more pure dingoes and high-dingo hybrids in the north west of New South Wales and in northern South Australia than in south eastern and eastern samples (Fig. 1). Within the WV population, all samples provided usable DNA, and all exhibited between 62 and 86% dingo ancestry (Table 1). No pure modern dogs (i.e. ≤ 10% dingo ancestry) were detected in the WV sample.

**Table 1:** Results of dingo ancestry testing for all individuals (Count—all) and for only the Western Victorian individuals (Count—WV). No WV hybrids were < 60% dingo.

Percentage (%)	Count—all	Count—WV
90–100	340	0
70–89	563	46
50–69	209	13
30–49	15	0
11–29	4	0
0–10	7	0

Eighty individuals with less than 60% dingo DNA were removed from the wider sample for subsequent analyses to reduce the noise from modern domestic dog DNA (whose genetic history is uncoupled from the local geographic movement patterns) but still retain good coverage of the study area, including all the WV individuals.

### Population structure

**Figure 2:**
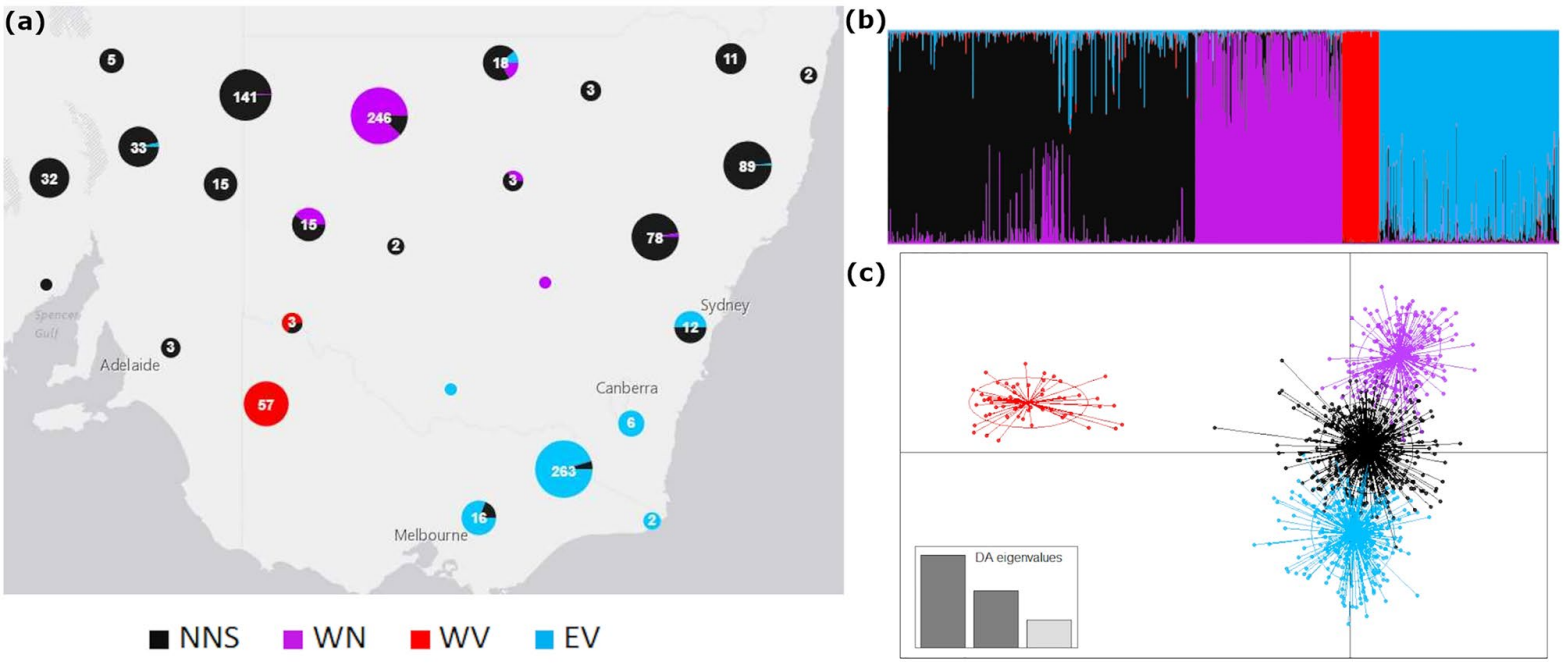
STRUCTURE and DAPC analyses of 1058 free-ranging dogs showing four clusters and genetic isolation of the WV population. Populations are coloured according to location: *NNS* Northern New South Wales and South Australia, *WN* Western New South Wales, *WV* Western Victoria and *EV* Eastern Victoria. (**a**) Results of STRUCTURE analysis, circles represent the proportion of assignment of each genotype to each cluster combined per location to better visualise samples collected at the same site. Numbers in white are the number of samples represented within each pie chart, which is also represented by the size of each chart (**b**) Bar plot of STRUCTURE results, each vertical line represents an individual coloured by the proportion of their genotype assigned to each cluster. (**c**) DAPC scatterplot of clusters showing isolation of the WV population. Eigenvalues for the discriminant analysis are presented in the bottom left of the plot.

From the Structure results, inspection of ΔK and lnPr(k) indicated K = 4 as the most likely number of clusters, dividing the sample into clusters from eastern Victoria, eastern New South Wales across to South Australia, western New South Wales and Western Victoria (Fig. 2a, b). Thermodynamic integration in rMaverick showed a maximum at six clusters (Supplementary Fig. S1). The greatest decrease in BIC output for assessing the number of clusters for DAPC were for K = 4–6, with a sharp decrease until K = 4, then slower decrease until K = 6 with 150 principal components retained (Supplementary Fig. S2). Ward’s clustering indicated optimal K of 6. At values above K = 4 the structure replicates were inconsistent and DAPC clusters were overlapping on the Y-axis. The Evanno method of determining K (∆K) captures the highest level of population structuring in the data, and inspection of the Structure bar plots for K = 6 indicates that there is sub-structuring within the northern New South Wales/South Australia (NNS) population (dividing at the approximate location of the western NSW population, WN) and within the WN population (splitting into a north and south population) (Supplementary Fig. S3). K = 4 is presented here as the focus is on the WV population rather than the surrounding areas. Both values of K showed the same trends in the summary statistics and absence of admixture in the WV population.

Inspection of the replicates for both K = 4 and K = 6 from the Structure iterations showed nine replicates with very similar results and one replicate with some differences in assignment (the major and minor modes^23^). As the nine similar replicates contained the runs with the highest log likelihood, these nine were aligned and averaged and the outlier replicate removed.

Cross-validation of the DAPC indicated an optimal number of principal components of 20 with missing data replaced by zeroes, and 30 with replacement with mean allele frequencies. The number of PCs retained was 25, and all three eigenvalues (Fig. 2c).

To establish whether the population structure identified was being influenced by introgression between dingoes and domestic dogs as well as location, we repeated the Structure analysis for K = 1–10 and the DAPC with 34 loci and the dingo and domestic dog reference individuals included. The Structure analysis showed an optimal ∆K = 2 (Fig. S4a), separating the dingoes and domestic dogs at the highest level of population structure (Fig. S4b). The DAPC analysis showed the same pattern as the DAPC in Fig. 2c for the populations included in both analyses, with the dingoes clustering with the EV/NNS/WN populations, the modern domestic dogs separated on X-axis, and the WV individuals separated on the Y-axis (Fig. S4c). These results indicate that the population structure is occurring on a level of hierarchical structure below the separation of dingoes and dogs.

### Genetic diversity statistics

Two individuals from the north of the WV area clustered with the 57 WV specimens, bringing the size of the WV population analysed for population summary statistics to 59. The WV population showed lower genetic diversity than the other populations on all population summary statistics tested, including allelic richness, which accounts for the difference in sample size, but did not have a higher number of private alleles (Table 2). Mean relatedness values were significantly higher than zero in all populations (zero representing random mating across populations), but the WV population showed mean relatedness over three times higher than the next highest population (WN). Due to the low genetic diversity and potential inbreeding in this population, this measure may not represent kinship in the strict sense, but is a further reflection that a high number of alleles are identical, presumably by descent from founding (if there was a small founder population) or surviving individuals (if there was a later bottleneck event). Effective population size was lower in the WV population than the NNS and EV populations, although similar to the WN population (Table 2). Both the WV and WN populations cover a smaller geographic area than the other two populations.

**Table 2:** Allelic patterns across four Structure-determined free-ranging dog populations showing lower genetic diversity in the WV population.

	EV	NNS	WN	WV
NI	283	485	231	59
Ar	6.7(6.24–7.12)	8.76(8.29–9.24)	5.46(5.09–5.79)	2.61(2.5–2.71)
PA	0.65(0.14)	3.03(0.45)	0.12(0.06)	0.09(0.05)
uHe	0.59(0.04)	0.6(0.05)	0.49(0.05)	0.31(0.05)
Ho	0.51(0.04)	0.51(0.04)	0.45(0.05)	0.26(0.04)
ML	0	0	0	9
MR	0.031(0.03–0.031)	0.006(0.006–0.007)	0.068(0.067–0.069)	0.241(0.237–0.245)
Ne	81 (77–86)	99 (96–103)	18 (18–19)	22(18–27)

*NI* number of individuals, *Ar* Allelic richness, *PA* number of private alleles, *uHe* Unbiased expected heterozygosity, *Ho* Observed heterozygosity, *ML* Number of monomorphic loci, *MR* Bootstrapped mean relatedness and *Ne* effective population size. Values in parentheses are standard errors, except for Ar and MR which is the 95% confidence interval about the mean calculated by 999 bootstrap replicates, and Ne is the parametric 95% confidence interval.

Pairwise differentiation, as measured by F_ST_, also confirmed strong genetic differentiation between the WV and all other populations, with the lowest value from the 95% confidence intervals of the WV comparisons (0.23 between WV and NNS) greater than the highest value from the comparisons between the other populations (0.15 between WN and EV) (Table 3). Jost’s D_EST_ showed the same pattern of differentiation as the F_ST_ (0.24–0.28 between WV and other clusters, 0.08–0.15 between the WN/NNS/EV clusters with no overlap of confidence intervals between the WV and others).

**Table 3:** F_ST_ values between the four populations determined by Structure results.

	EV	NNS	WN	WV
EV	*	0.055–0.065	0.13–0.15	0.28–0.30
NNS	0.060	*	0.07–0.08	0.23–0.25
WN	0.139	0.076	*	0.31–0.33
WV	0.291	0.240	0.321	*

F_ST_ values are below the diagonal, bias-corrected 95% confidence intervals from 1000 bootstrap replicates are above the diagonal.

**Figure 3:**
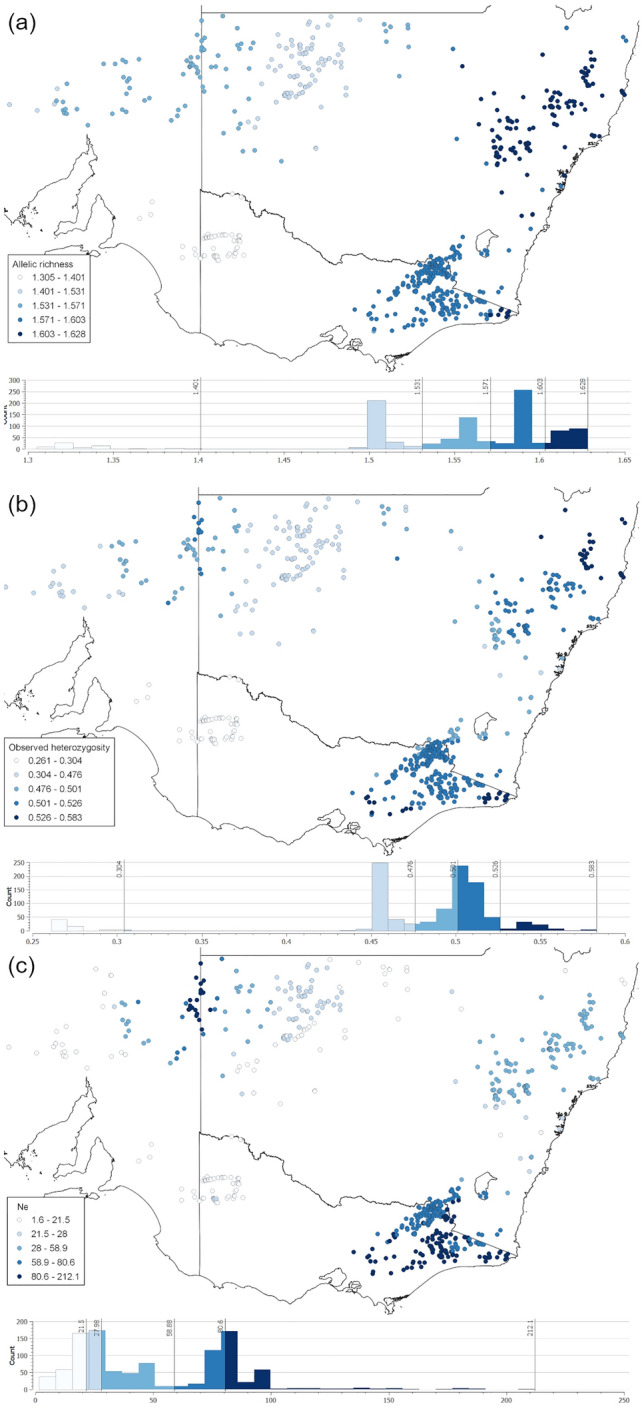
*sGD* values for each individual plus individuals in the surrounding 200 km. Individuals with fewer than 10 surrounding points within 200 km are not shown. (**a**) Allelic richness values, graded from lightest blue for low values to dark blue for higher values using five Jenks natural breaks, calculated and mapped using QGIS V3.22.3 (http://www.qgis.org). Histogram of allelic richness values distribution from QGIS is shown below the map; vertical lines indicate the values of the Jenks breaks. (**b**) As for (**a**), showing observed heterozygosity. (**c**) As for (**a**), showing effective population size calculated using alleles with frequency > 0.02.

The results of the *sGD* analyses support the results of the population-level, non-spatial analyses with respect to the WV population (Fig. 3). Observed heterozygosity (Fig. 3a) and allelic richness (Fig. 3b) measures were notably lower in the WV population area. The mean number of individuals included in each calculation was 180.2 (range 10–364, SD = 84.1). Effective population size (Fig. 3c) showed a general pattern of being higher at the coast and lower inland, excepting samples near to the New South Wales/South Australian border. Most individuals had values < 100, indicating overall low effective population size in dingoes.

## Discussion

Our southern Australian sample showed a similar pattern of dingo hybridisation to other studies, with higher numbers of hybrids near the more densely human-occupied coastal areas and higher dingo ancestry in the sparsely populated inland^3,17^. Similarly, pure modern domestic dogs were very rare. The WV population displayed a high level of dingo ancestry and isolation. It is possible that both are interrelated, with the large area of remnant mallee woodland in Big Desert and Wyperfeld National Parks providing a refuge for an already isolated dingo population, and the low human population density reducing the rate of ingress of modern dog genes into the population. Current patterns of modern domestic dog introgression in dingoes may not be possible to reverse without intervention by humans, where high dingo content hybrids and pure animals are captured, tested, and retained and backcrossed with pure animals, and other hybrids are culled. One process for reversal of hybridisation is captive breeding from “pure” stock, as recommended for the endangered wildcat (*Felis silvestris* ssp.^24^). It is unlikely that such direct dingo conservation processes will be introduced into the region because of extant livestock protection imperatives and the legal and animal welfare obligations of livestock producers. However, the current Victorian policy of limiting control activities on public lands to within 3 km of the edge (Agriculture Victoria website, https://agriculture.vic.gov.au/biosecurity/pest-animals/managing-wild-dogs-in-victoria) could slow the rate of hybridisation by reducing the probability of interbreeding between extant high-dingo hybrids and lower dingo hybrids from elsewhere. This suggestion is contrary to the findings of Bohling and Waits^25^ that lethal control increased hybridisation between red wolves (*Canis rufus*) and coyotes (*C. latrans*) and the suggestions of Cairns et al*.*^17^ that lethal control increases hybridisation of dingoes and other dogs. Data on the impact of lethal control on dingo hybridisation is lacking, so there is still much to be learned about these processes.

Hybridisation proportions might also correlate with expansion of livestock production and associated water infrastructure^26^. Conversion of ephemeral water supplies to permanency is only recent in the Mallee and Wimmera regions of Victoria. There is no permanent surface water drainage in the Mallee and most water availability was ephemeral prior to the introduction of a channel system over the 50 years from 1856^27^. Indigenous land use practices could also have affected dingo population density and distribution before British colonisation, but permanent water availability would have had influence on their distribution and that of their prey (dogs need between 26.2 and 122.6 mL kg^-1^ day^-1^ of water)^28,29^. It is likely that pre-British dingo population densities were low in WV because it is semi-arid (250–400 mm mean annual rainfall) and has low primary productivity due to poor soils. Any expansion of dingoes’ range due to a sudden increase in resources, coupled with the rapid spread of modern domestic dogs and associated mating opportunities is likely to have had conflicting effects on the genetic composition of dingoes: a decrease in genetic diversity in areas where rapid expansion has occurred due to small groups colonising newly hospitable regions, and an increase in genetic diversity where domestic dog genes were entering the population.

Although the population-wide proportion of dingo ancestry was high in the WV population, we are cautious about the finding that there were no ‘pure’ dingoes in the sample. The method of testing relies on updating of the model priors from reference populations, and sufficient deviation in allele frequencies from those represented in the reference populations is likely to decrease the accuracy of dingo ancestry estimation. The genetic diversity of dingoes across Australia is low overall^3,11^, which makes it easier for the reference population to capture the allelic variation in most dingoes, but unusually isolated populations such as WV and K'gari may show decreased accuracy with any reference sample approach. The DAPC analysis with the dingo and domestic dog references included (Fig. S4c) confirms that the WV population allele frequencies are distinct from the dingo and modern domestic dog reference populations, and that WV individuals are unlikely to be accurately represented in any method of ancestry analysis which requires such references. Although the number of private alleles was not high in the WV population, which would mean there is no analogue in the reference animals, the low genetic diversity and skewness from the references mean the comparisons are more susceptible to stochastic allele changes from genetic drift and the impact of data scoring errors.

In the short term, an assessment of the effect of reference population selection and quantification of the genetic variation of dingoes across their range is required to fully understand these impacts. In the longer term, expanding genome coverage should better characterise isolated and distinctive populations such as WV (e.g.^30,31^). A major advantage of microsatellite genotyping currently is the ability to add new samples to a dataset containing many thousands of already typed individuals^3^, and it will be some time before the availability of genomic data sets can provide similar baseline data. Expanding the genomic coverage of markers can also provide opportunities to clarify the history of the WV population. The increased resolution afforded by resampling a sub-set of the data with single nucleotide polymorphisms (SNPs) should allow much greater insight into the timings of population separations^32^. Our study also did not provide a clear indication of which of the surrounding areas was most closely related to the WV population, and higher-resolution markers may help to address this question.

The geographic limits of the WV population have not been determined by this study; further sampling in the surrounding areas would enable a greater understanding of the extent of this population, and the local environment that has enabled the isolation of these dingoes despite the dispersal capacity of free-roaming dogs^33,34^. Two individuals from the 2015 data collected north of the WV site clustered with the WV specimens in our study. However, the two individuals were not submitted with geographic coordinates, so their location is approximate. Although the presence of free-roaming dogs may be sparse in the surrounding areas, our study contains dingoes and hybrids caught in areas of South Australia marked as “not known to be occupied” by free-roaming dogs in a major 2013 survey^14^. Consequently, there is potential to improve our understanding of this region by sampling to the north and west of the WV population. There is also the potential for landscape analysis of co-correlates with population structure, such as land use, vegetation type, faunal assemblages and resource availability, to quantify potential barriers to gene flow in dogs that may help to identify areas where other isolated populations could occur.

The individuals across most of the study area displayed a pattern consistent with weak or clinal population structure: low F_ST_ values combined with evidence of admixture between all clusters in the north and east of the study area. The WV population displayed a distinctly different pattern, with high genetic differentiation, as demonstrated by both the F_ST_ and DAPC results, low genetic diversity and no evidence of admixture of individuals within the WV area; this implies sustained isolation of WV from surrounding areas. The differentiation, as measured by F_ST_, was considerably greater between the WV and other populations than would be expected for its distance from the other samples. The F_ST_ between the other populations tested were mostly around 0.1 (range 0.06–0.139), but all values between the other populations and the WV population were 0.240–0.321, indicating strong genetic discontinuity between WV and the surrounding populations, even over larger geographic distance than between the other populations (e.g. there is approximately 600 km between the centre of the WN and WV clusters, and approximately 850 km between WN and EV). The *sGD* results also show lower individual values for observed heterozygosity and allelic richness in the WV than any other area, which supports the results of the partitioned summary statistics and provides further evidence for the isolation of the WV population.

The effective population size, by contrast, is not uniquely low within the WV, with individuals in western New South Wales and in South Australia showing similar values. Broader comparisons of effective population size in high dingo-ancestry populations, particularly in inland, north and western Australia where high numbers of ‘pure’ dingoes are available, could indicate the typical effective population size for Australian dingoes, and their capacity to persist with low genetic diversity when dog introgression is minimal.

The presence of low genetic diversity, high mean relatedness and, in particular, the low incidence of private alleles is consistent with a population that is a reduced version of a larger regional gene pool and subject to drift and inbreeding—remnant after ~ 150 years of intermittent control in the region—rather than a population translocated from a different ancestral source^35^.

The WV population of free-ranging dogs shows measures of genetic diversity close to those found in the dingo population on K’gari^36^. Allelic richness, observed heterozygosity and effective population size values were similar (Allelic richness = 3.61 in K’gari and 2.61 in WV, observed heterozygosity = 0.27 in K’gari and 0.26 in WV, effective population size 25.7 in K’gari and 22.0 in WV), with the K’gari study using a reduced version of the same microsatellite marker set employed in this study. Although low genetic diversity due to founder effects, inbreeding and genetic drift are common in island populations^37,38^, finding comparable levels of isolation in a mainland dingo population was unexpected.

The reasons for genetic isolation in a large mobile apex predator in the face of an increasingly hybridised swarm require investigation. Prior to British colonisation and settlement, indigenous population density and their trading and cultural movements likely influenced the spread and continental distribution of dingoes. Biogeographic barriers to human and animal movements possibly affected colonisation by dingoes and subsequently impeded gene flow through biogeographic semi-isolation. Previous analyses of genetic subdivision in dingoes focussed on either a broad geographic range with few samples^4,11^ or a more limited area with more intensive sampling^16,36^. The Australia-wide studies identified a split between the lineages of dingoes in the south east and north west. The population structure analysis in this study, which is the first to characterise the west of NSW and Victoria, did not identify a contemporary boundary of the south east-north west split^4,11^. At the highest level of population structure (K = 4) the north of the study area showed a continuous population from eastern NSW to South Australia, with the exception of the WN population, which appears to exist within this continuum and may be the result of local environmental factors or a migration event. A discontinuity may still be present to the west or north of the area studied here, but this requires further investigation. Although the northern population is not the focus of this study, it further highlights the need for adequate sampling of dingo populations to disentangle their complex history of migration, colonisation, hybridisation, persecution and translocation which has occurred in the very short time, in evolutionary timescales, since their arrival in Australia.

Sampling in inner-eastern Australia is necessary to understand the contemporary and finer-scale processes at work in this separation, but the current study highlights an important caveat; sampling of individuals in the WV as representative of this area would provide an inaccurate genetic profile of the broader area. The high mean relatedness and lack of admixture with surrounding individuals could appear as a distinct lineage under some analyses, and care should be taken to examine demographic parameters for inbreeding and outbreeding that may be caused by isolation or introgression, respectively.

Although the south east/north west split and the K’gari subdivision have previously been combined under ‘ESUs’^4^, subsequent analyses suggest the processes that caused them differed. The K’gari (and WV) populations appear to have arisen from isolation after the colonisation of the continent by dingoes^36^, while the mitochondrial south east/north west lineages are more likely to have occurred during their initial or early migration into Australia, with unknown contemporary ecological significance. We should therefore consider more subtle effects across the landscape given complex but temporally shallow demographic history of dingoes, to then relay this information to local managers to enable more precise recommendations in the context of regional conditions and pressures.

The fate of the Australian dingo is a concern for many stakeholders across its broad and varied range^39,40^. Many questions remain about their introduction, ecology and behaviour, and the use of molecular data will continue to elucidate aspects of dingo distribution, subpopulation structure, and lineages nationally. This information will assist in the management of dingoes so that their genetic diversity can be maintained in the context of necessary control of livestock predation^15^ and conservation of predation-susceptible fauna (e.g. Olive Ridley turtles, *Lepidochelys olivacea*^41^ and northern hairy-nosed wombats, *Lasiorhinus krefftii*^42^). Here our objective was to add to that general body of work by detailing the genetics of one isolated population of dingoes. We hope it will fit into a broader context of studies into free-ranging dog genetic diversity across the continent and significant island populations of dingoes such as that on K’gari, utilising different genetic markers, analysis techniques and spatial scales of interest^11,43^.

## Methods

### Study region

The Mallee region of north-western Victoria is sparsely populated by people and contains a large area of unoccupied mallee (*Eucalyptus* spp.) woodland as national park (gazetted in 1909) and forestry lands. The region is part of the Murray-Darling Depression bioregion^44^, is semi-arid with mean annual rainfall of 300–320 mm^45^ and characterised by sandy soils of low fertility and productivity^46^. Agricultural enterprises, primarily cropping with some livestock production (mostly sheep at low carrying capacity), surround the mallee woodland. This geographic arrangement contributes to conflict between sheep graziers and free-ranging dogs, and the region is subject to ongoing free-ranging dog management by lethal control through trapping and baiting. The Wyperfeld/ Big Desert Wild Dog Management Zone employed five registered private trappers during the study, and contractors in contiguous nearby South Australia were responsible for controlling errant dogs. Together, these areas comprised the WV sampling region.

### Sampling

**Figure 4:**
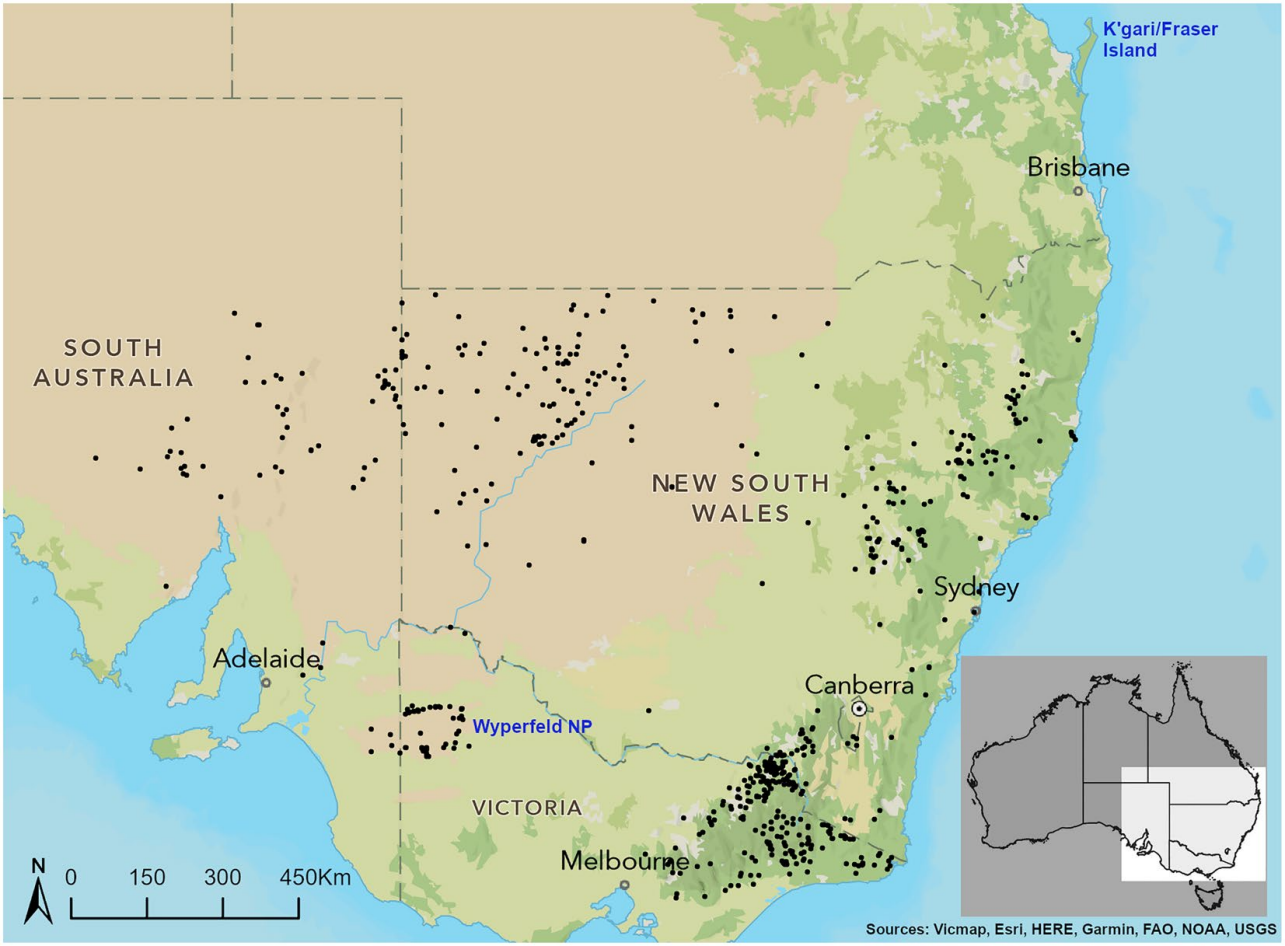
Locations of 1138 samples (black dots) taken from controlled free-ranging dogs. Capital city names are in lower case black text, state names are in capitals. The Wyperfeld/ Big Desert Wild Dog Management Zone and adjacent South Australian capture area (Western Victoria, n = 59) is noted in blue text, as is K’gari/Fraser Island which contains a comparable population. Dingoes and other free-ranging dogs are largely absent from the blank areas of New South Wales, Victoria and South Australia, but they are present and unsampled in the blank areas surrounding Canberra and coastal north-eastern and south-eastern NSW. Dashed lines show state borders, inset map shows the extent of the map within Australia in light area. Map was created using ArcGIS Pro v2.9.3 with terrain from “Outdoor” [basemap] Scale Not Given. June 16, 2022. https://www.arcgis.com/apps/mapviewer/index.html?webmap=2e8a3ccdfd6d42a995b79812b3b0ebc6 (accessed July 19, 2022).

Over 6.2 years, 57 samples were collected from free-ranging dogs killed by the trappers in WV from July 2012 to September 2018. This collection was part of a bigger and opportunistic sampling regime conducted between 2002 and 2020 in south-eastern Australia by authors PJSF, ES and DS, and collaborators from South Australia, New South Wales and Victoria comprising 834 samples. An additional 304 genotypes selected from Dryad (https://doi.org/10.5061/dryad.2rd32) were also included (Fig. 4). Excepting some areas in the Australian Capital Territory and coastal north-eastern and south-eastern New South Wales sampling coverage closely aligned with the extant distribution of free-ranging dogs^14,15^.

All wild dogs were either killed by third parties during obligatory wild dog management programs in Victoria, New South Wales and South Australia and so were dead at time of sampling, or were collected from live animals as part of research programs under the animal research authorities of the Orange Animal Ethics Committee, ORA06/009, ORA 12/15/024 and ORA 15/18/002, which were issued according to the *NSW Animal Research Act 1985* and following the recommendations in the ARRIVE guidelines.

### Laboratory protocols

Dog ear tissue specimens were stored in ~ 3 mL Longmire buffer^47^ until DNA extraction. Extractions were performed using Qiagen DNeasy blood and tissue kit (Qiagen Inc. CA, USA) with the recommended protocol. Extracts were amplified at 34 microsatellite markers in seven multiplexed PCRs^3,48^. PCRs were performed in 10 µl reactions, containing 5 μl Qiagen Multiplex PCR solution (Qiagen Inc. Valencia, CA, USA), 1 μl Qiagen Q-Solution, 1 μl DNA, 0.2 μM of each primer and 2 μl DNAase/RNAase-free water. PCR conditions were: 15 min at 95 °C, 35 cycles of 30 s at 94 °C, 90 s at 55 °C or 58 °C and 60 s at 72 °C, then 30 min at 60 °C. All fragments were visualised on the same equipment and peaks called by the same individual for consistency.

### Population analyses

Dingo purity testing was carried out on all samples using the method outlined in Stephens et al.^3^ 23 microsatellite loci^18^ were analysed in STRUCTURE v2.3.4^49^ along with 322 reference dingoes and 109 domestic dogs^3^. Reference dingoes were collected from across Australia, and although higher numbers from the central and west were used, repeating the tests with different references showed mean differences between results of < 1%^3^. Reference animals were coded with USEPOPINFO = 1 and test animals with USEPOPINFO = 0 (the ‘learning samples’ approach^49^). The sample was run for 300,000 iterations and 30,000 burn-in replicates, with allele frequencies initialised and updated from the reference individuals. Ten replicates were performed and the results combined using the R package *pophelper* v2.3.1^50^. The assignment of each individual’s genotype to the dingo reference population was then reported as a percentage. Individuals with less than 60% dingo ancestry were excluded from further analyses, to maximise the geographically relevant information in the genotype.

The remaining samples were then assessed for population differentiation using all 34 loci. The additional 11 loci were added to the 23 loci used for dingo ancestry testing to mitigate potential bias introduced by the selection of the 23 loci for allele frequency differences between dogs and dingoes. Each sample was analysed using STRUCTURE using the admixture model and correlated allele frequencies for 500,000 iterations and 50,000 burn-ins. The sample was run with initial Alpha = 0.25 and allowed to vary among populations^51^. Runs were repeated from K = 1–15 for 10 replicates. STRUCTURE output was assessed for optimal number of clusters (K) by the ΔK method^52^, by inspection of lnPr(K) using Structure Harvester v0.6.94^53^ and using the thermodynamic integration approach with the admixture model implemented in *rMaverick* v1.0.5^54^. The latter method provides an alternative estimate of K from multiple MCMC chains without assuming normal distribution of the likelihood. Replicate runs of the optimal K were then aligned, averaged and visualised using *pophelper*. Individuals were assigned to the cluster to which they had the highest ancestry coefficient (Q-value).

Populations were also assessed using Discriminant Analysis of Principal Components (DAPC) in R using the package *adegenet* 2.1.3^55,56^ which provides an assumption-free method for comparison with the Bayesian clustering approach. The number of clusters was inferred using the find.clusters function^56^ for K = 1–20 and inspection of the BIC graph for the minimum values. Optimal K value was also informed by the Ward’s clustering algorithm implemented in *adegenet* (criterion “diffNgroup”). The number of principal components to retain was chosen using the cross-validation method in *adegenet*, with missing data replaced with either zeroes or the mean of allele frequencies and the results compared.

### Population summary statistics

Population summary statistics were calculated for each of the STRUCTURE populations to characterise the relative genetic diversity within and between each population. The per-population allelic richness was calculated using *diveRsity* v1.9.90^57^. The number of private alleles, unbiased expected heterozygosity, observed homozygosity and bootstrapped mean relatedness were calculated using GenAlEx v6.51b2^58,59^. Mean relatedness values were calculated from the matrix of Lynch & Ritland estimators^60^ between individuals then averaged per population. These data were bootstrapped and permuted for 999 replicates to obtain 95% confidence intervals (CI) and to test for significant deviation from the null hypothesis of average relatedness, respectively. Pairwise Weir & Cockerham’s F_ST_^61^ and D_EST_^62^ were also calculated using *diveRsity* and bootstrapped for 1,000 replicates to obtain 95% CI values. Effective population size was calculated using NeEstimator V2.1^63^ and the results are reported for the single-sample linkage disequilibrium method with random mating and alleles with frequency below 0.02 excluded, to reduce bias introduced by the inclusion of rare alleles.

Summary statistics were additionally analysed across the study area using the R package *sGD* v2.11^64^. This method does not assume pre-defined clusters to assess population statistics, but instead uses a user-defined radius around each individual to calculate properties such as observed heterozygosity and allelic richness. This method may reveal subtle genetic variations within populations, as well as being less reliant on assumptions of population boundaries which may be unclear in continuously distributed taxa such as free-ranging dogs. Based on previous extents of spatial autocorrelation in dingoes^16^ we selected a distance of 200 km across the study area of approximately 1500 km × 1200 km. Geographic coordinates were converted to UTM using *PBSmapping* v2.73.0 in R^65^. Although the study area covered three UTM zones, the majority of the individuals were collected in Zone 55, so this zone was selected by *PBSmapping* and the results inspected to ensure the individual locations were reasonably accurate. Observed heterozygosity, allelic richness and effective population size using NeEstimator V2.1 were calculated against a Euclidean distance matrix for individuals with a minimum of 10 other individuals within the radius. Individuals which did not have a minimum of 10 other individuals within 200 km were recorded as missing data.

## Supplementary Information

Supplementary Information.

## Data Availability

The datasets generated and analysed during the current study are available in the Dryad repository, https://doi.org/10.5061/dryad.k98sf7m83.
