# Supplementary material for: An isolated population reveals greater genetic structuring of the Australian dingo
Source: Sci Rep. 2022 Nov 9;12:19105. doi: 10.1038/s41598-022-23648-1 (PMC9646726; doi:10.1038/s41598-022-23648-1)
Supplement: Supplementary file 1 — Supplementary Information. [file 41598_2022_23648_MOESM1_ESM.docx]

Supplementary Information


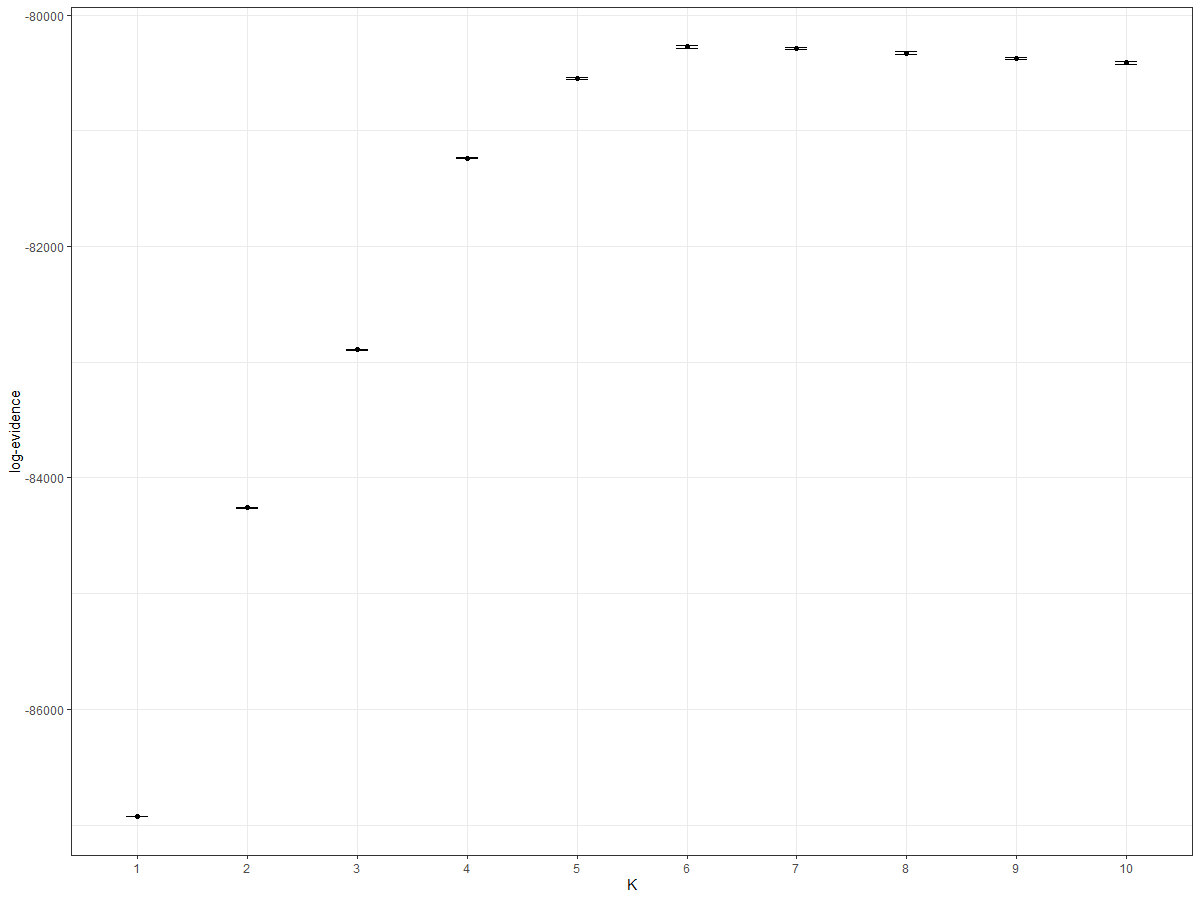


Fig S1: Thermodynamic integration plot from rMaverick


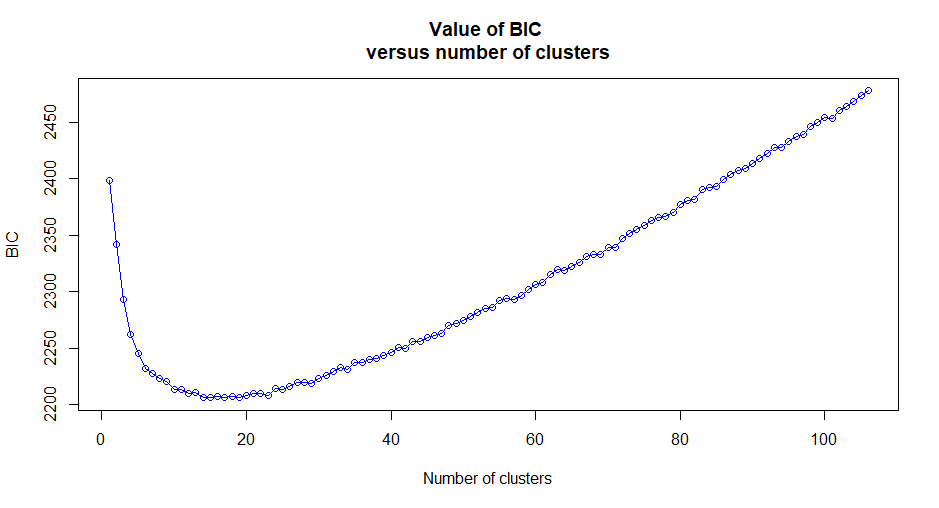


Fig S2: Bayesian Information Criterion (BIC) plot


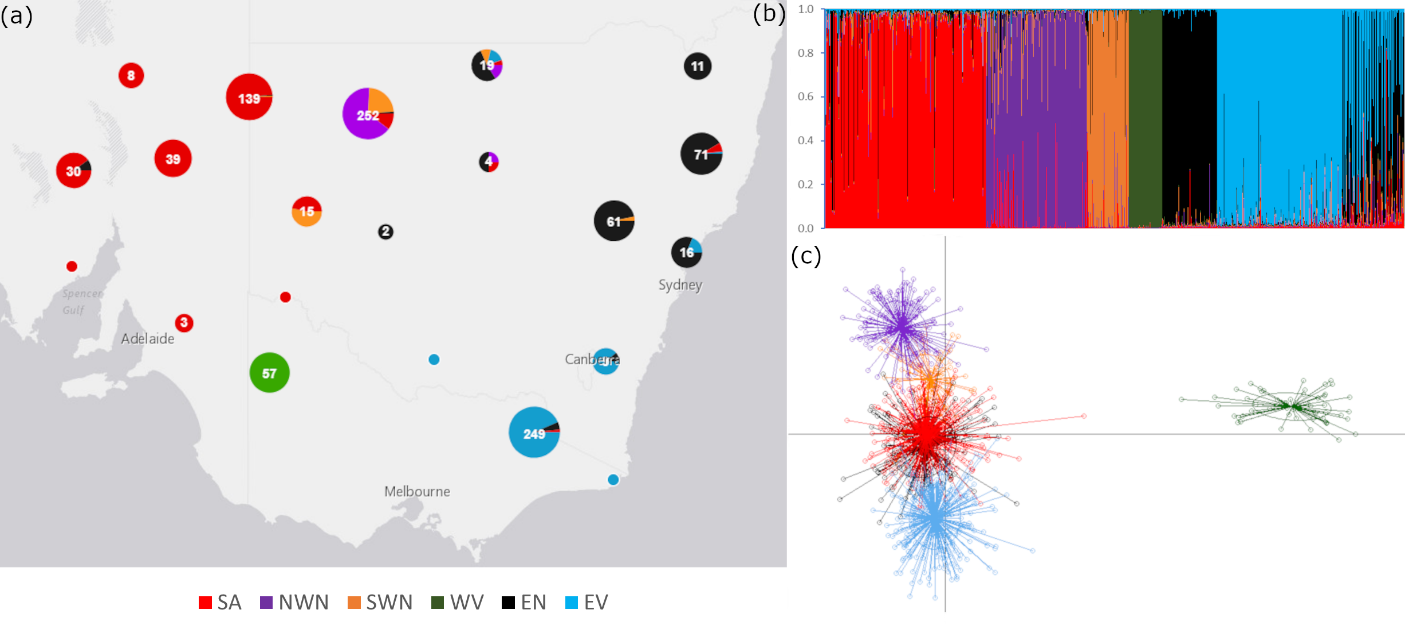


Fig S3: Structure (a-b) and DAPC (c) results for K=6. Population abbreviations are SA= South Australia; NWN= North west NSW; SWN = South west NSW; WV = Western Victoria; EN = Eastern NSW and EV = Eastern Victoria.


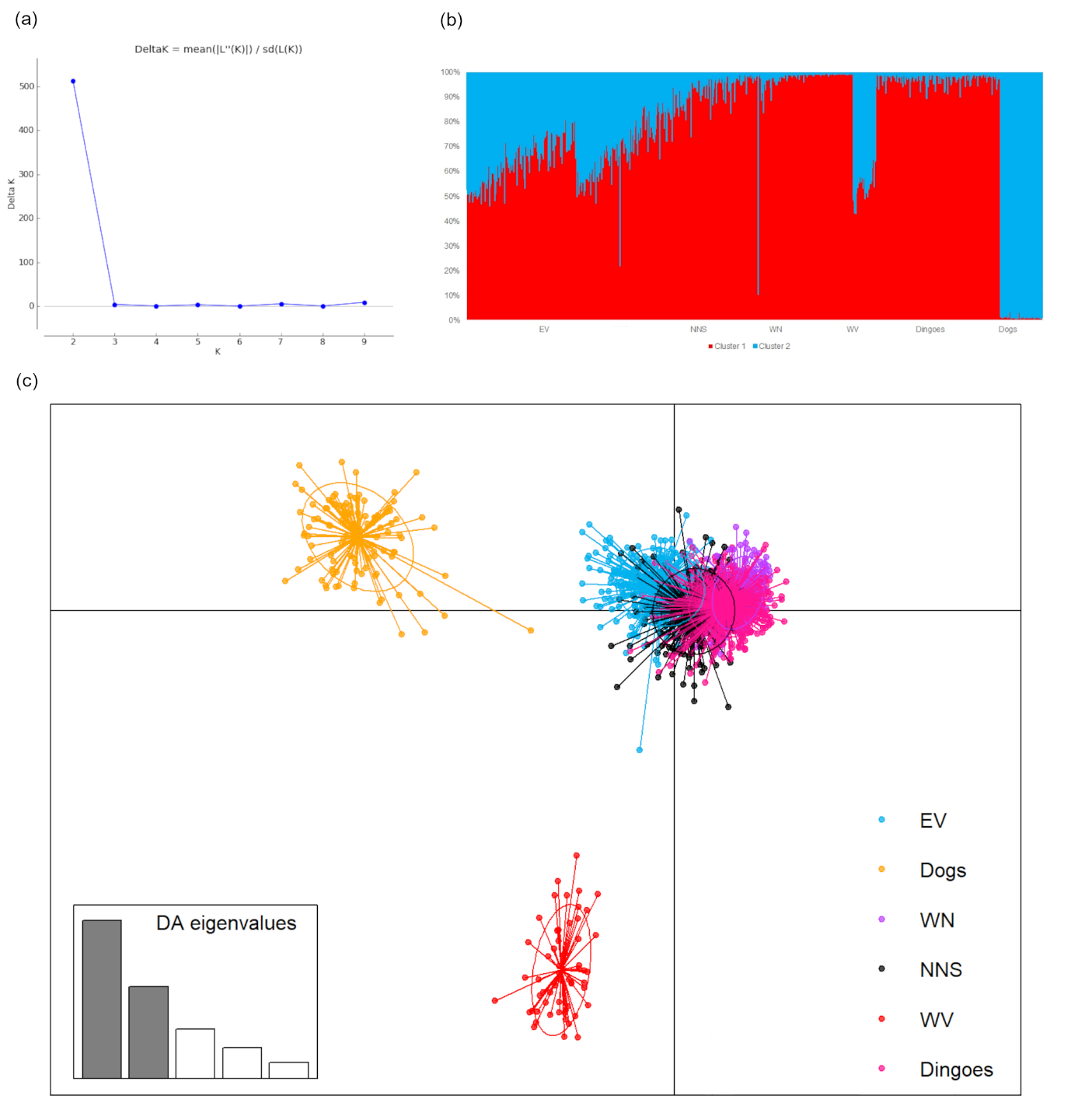
Fig S4. Results of STRUCTURE and DAPC analyses for all individuals sampled, plus the dingo and modern domestic dog reference individuals used for ancestry testing. (a) Results of ∆K testing for K=1-10 with 10 replicates, showing optimal K=2. (b) Structure barplot of all individuals for K=2. (c) DAPC analysis for six clusters, showing separation of both domestic dogs and WV individuals from the overlapping clusters of the EV/WN/NNS and dingo reference populations.
